# The constraints and concerns regarding the size and/or shape of the second generation female condom: The narratives from the healthcare providers

**DOI:** 10.4102/phcfm.v8i2.1146

**Published:** 2016-05-31

**Authors:** Ramadimetja Mogale, Fhumulani M. Mulaudzi, Mmapheko D. Peu, Mamakwa S. Mataboge, Roinah Nagunyulu, Salaminah S. Phiri

**Affiliations:** 1 Department of Nursing Science, University of Pretoria, South Africa

## Abstract

**Background:**

Despite the redesigning of the Reality condom (FC) to a new version of the second generation female condom commonly known as (FC2), the users are persistently constrained and concerned about the size and shape of this new version. Condom use is aligned to the Millennium Development Goals (MDG) 3, 5 and 6, which address gender equality, improving maternal health and preventing HIV and AIDS.

**Aim:**

To explore and describe the constraints and concerns regarding the size and/or shape of the FC2.

**Setting:**

The study was conducted at Tshwane health district in Gauteng province.

**Methods:**

A qualitative exploratory descriptive design was used. Individual in-depth interviews that yielded narratives in a designated health district in South Africa were conducted.

**Results:**

From the analysis of narratives three specific themes emerged. Firstly, the specific theme was that the size and shape of FC2 is undesirable for the health care providers, which may lead women to contract HIV and AIDS. The second theme was that the size and shape of FC2 and female genitals makes insertion complicated and predisposes women to be vulnerable in sexual relationships. The third was that the size and shape of FC2 results in pain and discomfort during coitus, exposing women to unwanted pregnancies and HIV and AIDS.

**Conclusions:**

The findings indicated the need for an evocative collaborative, interdisciplinary ‘walk the talk’ sexual health and AIDS education training programme for health care providers in primary health care facilities. Such programmes, if maintained, may assist health care providers to achieve the MDG 3, 5 and 6.

## Introduction

The female condom has been in use for more than two decades, however its modification to a new version of second generation female condoms, commonly known as (FC2), still presents constraints for its use by women, including health care providers. The Reality (FC) and female condom (FC2) are similar in size, shape and colour.^1^ Both were designed to be one-size-fits-all. Ever since their launch these two female condoms were received with concerns about their appearances and the size ranges.^2^ However, the latter (FC2) was made of synthetic latex, which was developed to reduce costs of the former Reality (FC).^1^ Globally, FC2 has circulated in markets since 1993 but was only approved by the United States Food and Drug Administration in 2009.^2.^ The size ranges of these two female condoms are 170 mm and 180 mm in length, which is the same length as the male condom. The outer-ring diameters of both are 7.1 mm and 7.2 mm, whilst the inner-ring diameters are both 58 mm. The mid-body diameters are both 66 mm.^3,4^ Structurally, the two female condoms are almost similar in size and shape except for the material used for FC2.

There are also constraints and concerns regarding the male condom size. A study by^5^ reported that ill-fitting male condoms, due to the size, diminish sexual pleasure. Furthermore, the discourse is taken further by^6^ when citing that the male condoms may be either too small or too big to fit the penis of a particular man. In order to fit well, a male condom depends on the erect penis of that particular man, the elasticity of the rubber, the shape of the condom and the tightness of the rim.^2^ There is a need to address the concerns of the users of the male condom. The debate around the female condom, in this case the FC2, is that it is viewed as big and bulging by some women.^7^ Anatomically, the vagina is a fibro-muscular sheath which is like an ‘H’ that extends from the cervix to the external genitalia. It varies in dimensions and shape from woman to woman but is around 6.5 cm to 12.5 cm in length.^8^ The vaginal wall is lined with a series of ridges which are known as rugae. The rugae allow the vagina to either contract or expand to accommodate anything that passes through the vaginal canal.^8^ The bulging appearance of FC2 was made specifically to conform to the shape of the vagina. The following features contribute to the size and bulging appearance of the FC2. The FC2 is a soft, loose-fitting sheath designed to fit the anatomical shape of the vagina. Its descriptors are:

1. A sheath that lines the vagina that may partially and completely cover the external genitalia.
2. An internal anchor retention feature that facilitates the insertion, at the same time keeping the FC2 in situ.
3. An external anchor retention that prevents invagination and also serves to remove the condom after utilisation.

The discourse around the above-mentioned aesthetics leads to uncertainty in adopting FC2 and negative connotations for some users, including even the healthcare providers.^9,11,12,13^ The negative connotations create a sense of hesitancy to embracing the FC2, a situation which may predispose women to be vulnerable to unwanted pregnancies and HIV and AIDS.^9,10^ However, according to^2,6^ the size and shape are just relative constructs as they depend on the size of either the penis or vagina of the user.

Globally, the use of female condoms is undesirably low especially amongst women and adolescent girls.^9,3,6,13,14^ Various factors contribute to this limited use of FC2. In contrast to this trend, the United Nations Fund for Population Activities (UNFPA) reported a rise in the use of female condoms (FC2 included). A South African National Youth Risk Survey indicated that, in 2009, a total of 31% of sexually active female adolescents always used female condoms in their sexual encounters.^13^ Despite the results of a South African study that indicated 35.6% preference for FC2 as opposed to 16.3% of V-Amour and 47.5% of Woman’s Condom (WC),^14^ the users of FC2 are still not contented with the size and shape of this type of condom. Relevant to this study, the WC was rated better than both FC2 and V-Amour with regard to appearance, ease of use and overall fit.^14^

Health care workers have a duty to endorse the use of FC2 through counselling and distribution as a way of achieving the MDG 3, 5 and 6.^10^ However, Mantell et al.^13^ reported a missing link in the utilisation of condoms by the same health care providers who are the custodians of counselling and distribution of FC2. A recent South African study^13^ stated that health care providers, as the custodians of the use of female condoms, have a limited knowledge about FC2.^16^ This limited knowledge about the use of FC2 indicated frustration as health care workers’ endorsement had been reported scientifically as an essential factor for motivating their clients, especially regarding the use of condoms.^17,10^ The concern is that service providers’ personal testimonies and attitudes on FC2 might have a negative impact on the less enthusiastic condom users.^18^

Existing studies on acceptability of FC2^19^ indicate that a variety of factors contribute to the low rate use of the female condom. However, of importance is that this lowered rate is associated with the existence and persistence of the gender-based power inequalities in most sexual relationships.^14^ In addition to the gender-based inequalities in relationships, as reflected in the MDG 3, the design (thus the shape and size) is also a contributing factor to the limited use of FC2.^14,20^ This emphasises the fact that there are challenges with the use of condoms, including accidental breakage or slippage during coitus that may lead to unintended pregnancies. Many studies focus on the attitudinal and interpersonal obstacles to the use of FC2.^19^ In this body of research there is a gap in the information and scant data regarding FC2 size and shape from the perspectives of health care providers. This points to what the health care providers think and believe about the female condom as users, not as counsellors and distributors.

Regardless of the concerns about the shapes and sizes of the male and female condoms, there is limited data on how the size and shape influences the utilisation of FC2, especially by the health care providers.

### Purpose of the study

This article aims to explore and describe the constraints and concerns about the aesthetics of the FC2 as narrated directly by health care providers in Tshwane District, Gauteng province.

### Objective of the study

The objective of the study was as follows:

- To explore and describe constraints and concerns regarding the size and shape of the FC2 as narrated by the health care providers in Tshwane district, Gauteng province.

## Research methods and design

### Study design

The current research followed a generic qualitative research approach that used in-depth interviews to generate data. The in-depth interviews were conducted in English by the trained research assistant at the health care centres in Tshwane. The health care providers in this study work at the different health centres where they are responsible for maternal and child health promotion, including family planning and HIV and AIDS prevention.

### Study population and sampling

The participants for this study were health care providers working in Tshwane health district in Gauteng province. The study was conducted in the Tshwane health district in Gauteng that was comprised of:

- Two district hospitals, one in the city and one in a township.
- A Highly Active Antiretroviral Therapy (HAART) health provision site in the city.
- A gateway clinic in one district hospital.
- Three primary health care clinics with one clinic in an informal settlement.

All these health care facilities are offering HIV and AIDS prevention programmes. HIV and AIDS prevention programmes, through the HIV Counselling and Testing (HCT) policy, mandate and promote HIV testing and the use of condoms by all the clients as part of integrated primary health care.

All categories of health care providers were included in the study. These were professional nurses, enrolled nurses, enrolled nursing assistants, HCT providers and allied workers. HIV and AIDS care is one of the major components of their job descriptions, hence they were purposively sampled. Both sexes (male and female) were the probable participants. Female health care providers were the only participants in the final sample size. The participants in this study were comprised of operational Managers (*n* = 3), Professional Nurses (*n* = 15), Enrolled Nurses (*n* = 5), lay counsellors (*n* = 6); and an administrator (*n* = 1). See Table 1 for the characterisation of participants.

**TABLE 1:** Characterisation of the participants.

Variable	Sub-variable	Value
Gender	Male	0
	Female	28
Experience	< 10	14
	11-25	11
	26-40	3
	> 40	0
Category	Professional nurses	15
	Enrolled nurses	18
	Enrolled nursing assistants	3
	AIDS lay counsellor	1
	Administrator	5
	-	1
Healthcare facility	Primary healthcare facility	17
	HAART	8
	Post natal clinic	3

*Source*: Authors’ own work

### Data collection

Data were generated through the use of in-depth individual interviews. Narrative accounts were elicited explicitly through these in-depth interviews.^21^ All the in-depth interviews were recorded. As this was a qualitative research approach the generated data were continuously analysed and reflected upon. The in-depth interviews conducted yielded narratives on how the size and shape of FC2 poses concerns and constraints on the utilisation of FC2. Narratives as stories are seen and perceived as embodiments of people, hence they are embedded within the stream of experiences. Such narratives are personal in their own right as they uncover the phenomenon of interest.^22,23,24^ Moreover, the narratives are informative and powerful as, in real life, every person is considered to be a story with a repository for stories.^23^ In this study, the participants were taken as stories and storytellers, not just as the puzzle for research^25,23^ but as the people who provided the storied part of their ‘self’^26,23^ on the concerns and constraints regarding the size and shape of second generation female condoms.

The participants were given a choice on where the in-depth individual interviews were to be conducted. Most of the participants chose to be interviewed in their consultation rooms during tea time or lunch time. The health care providers were interviewed in the operational manager’s office as they, unlike enrolled nurses and enrolled nursing assistants, had no consultation rooms. The central question which was asked was: What do you think are the constraints and concerns regarding the size and/or shape of the FC2?

### Data analysis

The research team collaboratively and iteratively analysed the generated narratives after they were transcribed verbatim. The process of analysis started with a close reading of the narratives in order to identify different parts of the participants’ stories.^27,25^ The brief summaries of each participant’s narrative were prepared by first highlighting the general impressions and infrequent features of each story.^25^ Thereafter, each story was pictorially represented by a drawing drawn by each team member. The compiled summaries and drawings were compared through the consensus meeting.

The meetings assisted the team members to categorise central story lines^27,25^ from the participants about their concerns and constraints regarding the size and shape of FC2. Additionally, the consensus meetings helped the team members to identify the commonalities, differences and areas of importance within the story line.^27,25^ Such an analysis facilitated the emergence of important components of the story about the concerns with, and constraints of, the size and shape of FC2 used as a contraceptive method and an HIV and AIDS preventative strategy. Put differently, through the consensus meetings the common thread(s) that ran throughout the narratives about the shape and size of FC2 were recognised.^22^ Last, the continuous analysis and reflections shaped the authors’ understanding^23^ on the size and shape of FC2.

### Trustworthiness

Trustworthiness of the study was maintained and observed through credibility, dependability and transferability. Credibility was ensured by continuous engagement with the participants, and follow-up meetings were arranged to ensure sufficient time to collect data and to probe further. Data were collected over a month and every interview lasted 30-45 minutes. Setting triangulation was also achieved by including PHC setting, wellness clinics providing HAART and selected units in the hospital. This ensured that description of the size and shape of FC2 emerged as the health care providers were from different settings. The research methodology was thickly described so that any reader could derive her or his conclusion regarding the truth value of the study data in order to ensure transferability. Dependability was ensured by safe keeping of all the documents covering the process. The transcripts are available to any person who asks for them.

### Ethical considerations

The study obtained ethical approval from the University of Pretoria Ethics Review Board and the Gauteng Provincial Department of Health. Confidentiality and privacy of the study participants was maintained. All the participants signed informed consent after the researcher(s) went through the study information sheet with them. Privacy of participants was further ensured by assigning numbers or codes to the participants. Institutions included in the study were also not exposed by names. Additionally, all the research materials were kept securely in a locked cabinet located in an office and accessed only by the research team.

## Results

Through the continuous engagement with the narratives of the participants, the authors came to understand that the participants were very concerned with ‘the size and shape’ of FC2. As their engagement and reflection went deeper, the authors were able to understand why the shape and size of FC2 is important to the participants. By pausing and reflecting continuously on the general storyline, specific story lines emerged. First, the specific theme was that the size and shape of FC2 is undesirable for the health care providers. The second theme was that the size and shape of FC2 and female genitals makes insertion complicated. The third was that the size and shape of FC2 causes pain and discomfort during coitus.

### The size and shape of FC2 is undesirable to health care providers

Most participants described the size and the shape as a constraint to attaining pleasurable sex. Participant 13 (female, 54 years) said: ‘It [*FC2*] is not easily acceptable, the way it [*FC2*] is structured; the shape says no!’ Participant 2 (female, 36 years) said: ‘By [*just*] looking at it [*FC2*] was scary and even the shape looks horrible’. Additionally, participants indicated their concern about the two rings of the FC2. Participants commented that the rings made them hesitant and reluctant to try the FC2. Participant 20 (female, 45 years) described her thoughts the first time she saw the FC2: ‘I was like wow, how am I going to use this?’ Participant 23 (female, 48 years) said: ‘The rings looked scary, in fact the entire structure of FC2 is very scary’. Participant 1 (female, 48 years) indicated that the FC2 was not only scary but also ugly when she said: ‘I know the long tunnel one [*FC2*] that long ugly one [*FC2*]. I used it three years ago.’ To add to the disgust regarding FC2: Participant 4 added that:

‘If they can come with [*an*]other design the way the [*current FC2*] looks is very big and [*as women*] we are scared to introduce it to [*our*] partners with the fear … of failure. Not everybody can accept [*such a shape*].’ (female, 28 years)

### The size and shape of FC2 and female genitals make insertion complicated

Some participants indicated the challenges encountered when trying to use FC2 due to its size and shape. Participant 11 (female, 52 years) said, ‘Female condom is complicated especially the insertion procedure’. Participant 5 (female, 38 years) was of the same thought as she indicated that: ‘It [*FC2*] is complicated, I was unsure how to insert it … and it is difficult for one to understand if it has reached the cervix.’ When describing the size and shape of FC2, Participant 4 (female, 28 years) indicated that: ‘FC2s are not convenient, the way they [*FC2s*] are structured and the way [*women’s*] complex body is structured it is not easy to use them [*FC2s*] as compared to the male condom’. Participant 3 (female, 38 years) said: ‘The thought of the rings that goes inside and the other that stays outside put me off [*sexually*].’ Participant 6, (female, 39 years) confirmed: ‘Or maybe it is the posture, the sex organs of a woman which makes the insertion complicated.’

Another important issue which was raised by the participants was that the shape contributes to the complicated insertion procedure. Regarding this issue, Participant 2 (female, 36 years) expounded on this by saying that, ‘Generally insertion of FC2 is more complicated than male condom as it involves putting fingers into the vagina together with foreign objects.’ Participant 3 (female, 38 years) alluded to the complicated insertion by stating: ‘You must squat, make an 8, push up and 2 rings, one inside and one outside, this is too much information to comprehend; not everyone will understand it.’

The sense that was provided by the participants’ information was that they preferred male condoms over the FC2. This was indicated by Participant 17 (female, 52 years), who commented that: ‘[*With male condom*] it is evident when he puts it [*male condom*] on I can see it [*male condom*] and there is no way that it’ll go out’. Participant 4 (female, 28 years) said: ‘Like I’ve said, the posture, the sex organs of a woman, it is difficult for one to understand that I’ve reached the cervix. With male condom, the penis is straight, you just open insert and push back.’ Participant 8 (female, 33 years) confirmed that the male condom was easier to use and said: ‘The way they are structured and the way our complex body is structured it is not easy to use them as compared to the male condom.’ Participant 15 also supported the unacceptability of FC2 as she said:

‘[*Laughing*] ahem … I guess they introduced the male condom first and we got used to male condoms. Then later the female condom was introduced. People got this resistance especially because the procedure of insertion is too complex and its shape made people not comfortable of using it.’ (female, 32 years)

What might contribute to the non-use of FC2 is the lack of trust of partners. Participants indicated that due to the shape of FC2 there is a possibility of penile misrouting during coitus, hence Participant 7 (female, 48 years) said: ‘I fear he will deliberately go to the side and avoid entering inside the condom.’

### The size and shape of FC2 results in pain and discomfort during coitus

The participants discussed the size and shape of the FC2 being too complicated to be used. Participant 8 (female, 33 years) said: ‘I once have an interest of using it but it was challenging … for the first time it was a bit painful, the rings were hurting when I put it in.’ Participant 2 (female, 36 years) supported this statement by stating that: ‘It doesn’t fit correctly and it was painful during intercourse’. Participant 3 (female, 38 years) emphasised the discomfort of the rings by saying that: ‘It [*FC2*] was not so comfortable [*and this was*] because of the ring.’ Participant 24 reported the discomfort experienced by her partner, who did not want to use a male condom. She said:

‘I just told him I wanted to try the female condom because he always complained when I asked him to put on a male condom. But he also complained that he is not comfortable with it.’ (female, 26 years)

Participant 22 (female, 40 years) took the statement further saying that, ‘It makes noise; the friction is too noisy of which it is such an irritation.’ Another discomfort experienced by Participant 7 (female, 48 years) was that FC2 disturbed the spontaneity of sex. Participant 1 (female, 48 years) said: ‘It [*FC2*] is sort of on the way for foreplay and the spontaneity’ for sex. Participant 5 (female, 38 years) echoed the same sentiments: ‘By the way consented sex needs spontaneity for it to be intimate.’

Contrary to what some participants reported there were some health care providers who reported positively on FC2. Comments such as, ‘It is comfortable to use, you do not feel it during sex’ (Participant 9, female, 34 years), and:

‘There is a misconception going around with the use of female condom; that it is too big, they must make it smaller and try to make the shape look better which I don’t have problems with because when I used it there was no problem.’ (Participant 10, female, 54 years)

Participant 19 added saying:

‘They are fine; I don’t have a reason against them [*FC2*]. I only had the feeling that there is something inside my vagina. Actually I was just testing how it feels. I do give advices to people on how to use it. But all in all the experience was fine.’ (female, 38 years)

Some of the participants confirmed that they used FC2 to gain experience so that they would be able to promote its use amongst health care users. Participant 20 said:

‘No [*laughing*]. But I once tried it just to experience it. It need time, patient and competencies to master the procedure. You must be knowledgeable to use it and at my age, I don’t have time for that. Maybe I’m not patient enough. Because I just wanted to feel how my clients would feel when inserting it.’ (female, 39 years)

## Discussion

Currently, there are two types of female condoms most commonly used when not including the panty or bikini types used in the sex industry; in South Africa the most used type is FC2.^15^ When listening to these stories on the size and shape of FC2, the authors wondered if it was the issue of the size and shape or that the health care providers were reluctant, or not prepared, to try new things.^13^ The narratives of the size and shape indicate that there is a problem of health care providers giving information, education and counselling to clients on the method about which they lack pertinent details, such as size and shape of the FC2. Such lack of knowledge affects the minimal use of the condom at national and global level.^28^ Health care providers know that FC2, like other condoms, is not a natural object; there will always be discomfort when it is inserted.^14^ However, the discomfort felt with the use of FC2 led to the unacceptability of FC2 by some health care providers. It was, furthermore, a disappointing report from health care providers who compared the FC2 to male condoms and indicated male condoms were better and easier to use. These health care providers preferred male condoms as they feared males may deliberately misroute during coitus.^29,19^ Such doubts make the perception about FC2 negative and perpetuate the unacceptability of FC2 to health care providers. This stance may expose them to HIV and AIDS infections.^10^

The opposite position was also reported by health care providers, who reported to have positive experience during the use of FC2. They alluded to its use as a pleasurable experience with no discomfort.^3,30,19^ Such comments give hope to the promotion of FC2 by health care providers and gender equality in relationships where women would be able to negotiate for safe sex.^10^ The need to reach out to those who still lack information and skills to use FC2 cannot be underestimated. Health care providers who lack knowledge related to the anatomy of female genitals, specifically vagina and cervix, need to be provided with relevant teaching aids and condom education programmes so that they are enabled to use and promote FC2 in the context of the HIV epidemic and unwanted pregnancies.^32,10^ These authors further report that, during the study, participants requested condom demonstrations so they could understand how they should be used. However, there was a counter motion, citing the demonstrations would encourage premature sexuality.

The shape of FC2 was designed in relation to the anatomical structure of the vagina. Health care providers need to know this as the custodians of information, education and counselling on condom use, in this case FC2. It is a well-known fact that health care providers have the power of suggestive influence over clients.^13^ However, the lack of body knowledge noted in this study from health care providers was a major concern, as indicated by Mantell et al.^12^ One professional nurse who had studied biological science during her training indicated that they need to be shown how to use the female condom. In addition, she alluded to the fact that the information on the anatomy of the genitals in relation to the female condom needs to be provided. This implies that during female condom promotion the level of education of the health care provider should not be taken for granted. The relevant health care authority should undertake a thorough education of health care providers in promoting female condoms.

This study uncovered the normative beliefs such as the lack of enjoyment and pleasurable sexual encounters when using condoms, FC2 included, as cited by^18^. Additionally, the study revealed constraints and concerns about diminished intimacy and the spontaneity of sex as a result of the size and shape of FC2. The participants in this study were the people who provided information about FC2, concurrently endorsing its use, especially for HIV and AIDS prevention. It was strangely apparent that, in this era of HIV and AIDS, health care providers still have questions and concerns regarding FC2s size and shape.^13^ Furthermore, similar constraints and concerns communicated by health care users affected them personally and limited the use of FC2. The health care providers lacked experience of what they are expected to promote. Without lived experience they may be reluctant to promote the use of FC2 as they related that the shape is ugly and it is a foreign object. An evocative collaborative, interdisciplinary ‘walk the talk’ AIDS education training programme for health care providers would assist them in using the FC2 and in promoting its use to their clients. The ‘walk the talk’ programme is an experiential interactive training programme that will prepare health care providers to promote the use of FC2.^31^ According to Divaharan et al.,^31^ such a training programme involves experiential learning through the creation of practical learning experiences for the HCPs, and the use of reflections and reflective techniques during the training. Last, the programme is value driven and encourages a ‘practice makes perfect attitude’, simultaneously optimising the ‘change talk’ and reducing ‘sustain talk’.^28,14^ Health care providers need to accept FC2 use as a female empowering and HIV and AIDS prevention intervention.

### Recommendations

The participants were health care providers working in health care facilities mandated to offer an HIV and AIDS prevention programme. The type of health care facilities in this study have an HCT policy that mandates and promotes HIV testing, and the use of condoms by all the clients. By right, the staff members working in such facilities are employed based on their expertise in all aspects of HIV and AIDS prevention strategies. One such prevention strategy for women is the use of FC2. Who could have ever thought the ‘supposed to be reservoirs of knowledge’ lack pertinent knowledge and information on the shape and size of the FC2 in relation to that of the vagina? It is assumed that the participants in this study are the ones to have expert knowledge on the anatomy of a vagina in order to provide informed education, information and counselling to the clients on HIV and AIDS prevention.

## Conclusion

Regardless of the participants’ knowledge regarding the high rate of HIV and AIDS in South Africa unprotected sex as risky behaviour is still practiced. FC2 is the only dual barrier method for disease(s) and pregnancy prevention that provides women with sexual bargaining powers.
